# Potential Anticancer Activities of the Thai Traditional Medicinal Recipe Santakatpuakaln Against Colorectal Cancer Cell Lines

**DOI:** 10.1155/adpp/6682780

**Published:** 2025-10-28

**Authors:** Worrakanya Narakornwit, Roongtiwa Srisuphan, Uthai Sotanaphun, Pawaris Wongprayoon, Purin Charoensuksai

**Affiliations:** ^1^ Natural Products Research Center (NPRC), Faculty of Pharmacy, Silpakorn University, Nakhon Pathom, Thailand, su.ac.th; ^2^ Division of Industrial Pharmacy, Faculty of Pharmacy, Silpakorn University, Nakhon Pathom, Thailand, su.ac.th; ^3^ Faculty of Pharmacy, Silpakorn University, Nakhon Pathom, Thailand, su.ac.th; ^4^ Division of Biopharmaceutical Sciences and Pharmacology, Faculty of Pharmacy, Silpakorn University, Nakhon Pathom, Thailand, su.ac.th

**Keywords:** alternative medicine, anticancer, folklore medicine, herb, metastasis, traditional medicine

## Abstract

Traditional Thai herbal medicine has been used for centuries to treat various diseases, including cancer. One notable formulation, Santakatpuakaln (STK), has been employed to manage cancers, particularly of the gastrointestinal (GI) tract, such as liver and colorectal cancers (CRCs). Despite its historical use, scientific validation of its efficacy and safety remains vastly limited. In this study, we prepared an ethanol extract of STK and evaluated its anticancer properties in vitro. STK demonstrated significant cytotoxic activity against CRC cell lines (HCT15, NCI‐H508, HT29, and HCT116) and the liver cancer cell line HepG2 while sparing noncancerous colonic epithelial cells (FHC), suggesting a potential therapeutic window. The cytotoxic activity of STK was accompanied by apoptosis. Additionally, STK inhibited tumor spheroid growth and suppressed HCT116 cell migration in transwell assays, indicating both cytotoxic and antimigratory effects. These findings support the traditional use of STK in cancer management and warrant further investigation into its active compounds, mechanisms of action, and therapeutic potential.

## 1. Introduction

Colorectal cancer (CRC) represents a significant global health challenge, being the third most common cancer and the second leading cause of cancer‐related mortality [1, 2]. The burden of CRC is particularly pronounced in Western countries, where its incidence has been rising steadily due to factors such as an aging population, sedentary lifestyles, and dietary habits [3]. Current treatment options, including surgical resection and chemotherapy, have shown limited efficacy in enhancing long‐term survival rates [3, 4], underscoring the urgent need for the development of novel therapeutic strategies that target the underlying molecular mechanisms of CRC. Enhanced understanding of CRC pathophysiology and molecular pathways can facilitate the identification of new drug targets, thereby improving patient prognosis and reducing the overall disease burden.

The use of traditional herbal recipes (THR) for cancer management has a long‐standing history and is increasingly gaining recognition in contemporary medicine. These remedies, deeply rooted in ancient practices of various cultures, are believed to offer a range of benefits, including anti‐inflammatory properties, immune modulation, and the ability to target cancer cells [5, 6]. Scientific research has highlighted the anticancer potential of certain herbs, such as curcumin from turmeric, ginseng, and garlic, which are known to inhibit cancer cell proliferation and induce apoptosis [5]. Many cancer patients turn to THR as an alternative to avoid the adverse side effects of chemotherapy or use them concomitantly with modern anticancer therapies in hopes of enhancing therapeutic efficacy [6]. Notably, some THRs have a history of human use spanning centuries, lending them credibility in terms of efficacy and safety in traditional medicinal systems [6, 7]. These formulations often incorporate a balanced combination of herbal ingredients, following folklore medicinal principles designed to optimize therapeutic outcomes while reducing the side effects associated with conventional treatments [8, 9]. Despite this promising history, it is essential to approach THR with scientific rigor. Further comprehensive research in a well‐controlled environment is crucial to validate their efficacy, ensure safety, and uncover the mechanisms underlying their therapeutic effects in cancer management. This integration of traditional wisdom with modern science holds great potential for advancing cancer care.

The use of traditional medicine for cancer treatment in Thai culture, much like in other nations, has a rich history spanning centuries. Certain documented traditional Thai medical formulations for cancer treatment have been preserved in ancient scriptures and manuscripts over 200 years old. One prominent example is “Santakatpuakaln” (STK), a traditional Thai medicinal recipe specifically indicated for cancer treatment. The formulation of STK is inscribed in stone at Wat Phra Chetuphon Wimonmangkhlaram (commonly known as Wat Pho, coordinates: 13.667912769000951, 100.53660705151073) in Bangkok, Thailand, located at Pavilion 7, Pillar 7, Page 1 since the 1830s. Traditionally, STK has been used to treat abscesses in various organs, which were believed to cause “putrid blood” that could spread to other parts of the body, resembling the metastatic behavior of cancer. In traditional Thai medicine, certain cancers, such as those originating in the gallbladder, liver, lungs, heart, stomach, and intestines, were commonly referred to as abscesses. The 16 plant‐based ingredients of STK and the percentage used in the remedy were summarized in Table 1. For oral administration, all herbal components are pulverized into a fine powder. This powder is then encapsulated, with each capsule containing 500 mg. The recommended dosage is two capsules twice daily, administered orally before meals. An alternative method of delivery involves mixing the herbal powder with honey, which serves to enhance taste and ease consumption. STK is employed by traditional Thai healers to manage various types of cancer, particularly those of the digestive system, such as liver and CRCs.

**Table 1:** Summary of plant compositions, part used, and % w/w in STK remedy.

No.	Scientific name	Part used	% w/w
1.	*Acorus calamus* L.	Rhizome	2.17
2.	*Aloe* sp.	Concentrated and dried juice of the leaves of *Aloe* sp.	2.17
3.	*Angelica dahurica* (Hoffm.) Benth. & Hook. f. ex Franch. & Sav.	Root	2.17
4.	*Artemisia pallens* Wall. ex DC.	Aerial part	2.17
5.	*Avicennia marina* (Forssk.) Vierh.	Heartwood	32.61
6.	*Cannabis sativa* L.	Root, stem, leaves, and flower excepted fruit	2.17
7.	*Ferula assa-foetida* L.	Oleo‐gum‐resin	2.17
8.	*Gloriosa superba* L.	Tuber	2.17
9.	*Glycyrrhiza* sp.	Root and rhizome	2.17
10.	*Persicaria odorata* (Lour.) Soják	Aerial part	2.17
11.	*Piper nigrum* L.	Fruit	32.61
12.	*Piper retrofractum* Vahl	Infrutescence	4.35
13.	*Piper wallichii* (Miq.) Hand.‐Mazz.	Stem	2.17
14.	*Terminalia chebula* Retz.	Leaf gall	2.17
15.	*Typhonium trilobatum* (L.) Schott	Tuber	2.17
16.	*Zingiber officinale* Roscoe	Rhizome	4.35

Despite its long‐standing use in traditional practice, the efficacy and safety of STK have yet to undergo rigorous scientific validation, highlighting the need for evidence‐based research. In our study, through a series of in vitro experiments, we demonstrated that STK possesses anticancer activity against CRC cancer cells cultured under both 2D and 3D conditions. Our data thus provide a scientific basis supporting the anticancer properties of STK and pave the way for further research to explore its therapeutic potential.

## 2. Materials and Methods

### 2.1. Cell Lines, Chemicals, and Reagents

The cancerous cell lines HCT116, HT29, NCI‐H508, and HCT15 (colorectal); MDA‐MB‐231 (breast); HepG2 (hepatocellular carcinoma); HeLa (cervical); and the noncancerous fetal colonic epithelial cell line FHC were obtained from ATCC (USA). Cell culture media and supplements, including minimum essential medium (MEM), Dulbecco’s modified Eagle’s medium (DMEM), DMEM/F12, fetal bovine serum (FBS), Glutamax, nonessential amino acids (NEAA), and penicillin/streptomycin (Pen/Strep) solution, were sourced from Gibco (USA). Human epithelial growth factor (hEGF), insulin, transferrin, HEPES, dimethyl sulfoxide (DMSO), and 3‐(4,5‐Dimethylthiazol‐2‐yl)‐2,5‐diphenyltetrazolium bromide (MTT) were procured from Sigma‐Aldrich (USA). Tissue culture plates, including 96‐well, 6‐well, and U‐bottom ultralow attachment 96‐well plates (no. 7007), were sourced from Corning (USA). Transwell permeable supports with 8.0 μM polycarbonate membrane, 6.5 mm inserts for 24‐well plates were purchased from Costar (USA).

### 2.2. Plant Materials

Compositions of the Thai STK recipe, according to ancient scriptures written in Thai, are provided in Table 1. The resins of *Aloe* sp., the roots of *Angelica dahurica*, the aerial part of *Artemisia pallens*, the heartwood of *Avicennia marina*, the resin of *Ferula assa-foetida*, the tuber of *Gloriosa superba*, the root and rhizome of *Glycyrrhiza* sp., the aerial part of *Persicaria odorata*, the leaf gall of *Terminalia chebula*, and the rhizome of *Zingiber officinale* were purchased from an herbal drugstore in Bangkok, Thailand. The rhizome of *Acorus calamus*, all parts of the plant except for the fruit of​ *Cannabis sativa*, the fruit of *Piper nigrum,* the infructescence of *Piper retrofractum*, the stem of *Piper wallichii*, and the tuber of *Typhonium trilobatum* were purchased from villagers in Nakhon Si Thammarat Province, Thailand. Voucher specimens of all herbs were deposited at the herbarium of the Faculty of Pharmacy, Silpakorn University, Thailand. Pictures of each composition and specimen number are as listed in Supporting File: Supporting Table 1. Worrakanya Narakornwit identified all plant materials.

### 2.3. Preparation of STK Remedy Extract

Ten grams of STK remedy powder were macerated with 400 mL of absolute ethanol for 3 days at room temperature, then filtered, and the previous remedy powder was macerated again with the same procedure. After that, the filtrate was combined and evaporated with a rotary evaporator at 40°C under reduced pressure (approximately 90–100 mbar) to obtain 0.89 g of the extract. A portion of the extract was dissolved in DMSO to constitute STK crude extract and stored at −20°C for cell‐based assays.

### 2.4. Cell Culture

HCT116, HT29, and HepG2 cells were maintained in DMEM supplemented with 10% FBS, 1% NEAA, 1% Glutamax, and 1% Pen/Strep. HCT15 and NCI‐H508 were maintained in RPMI supplemented with 10% FBS and 1% Pen/Strep. HeLa was maintained in MEM supplemented with 10% FBS, 1% NEAA, 1% Glutamax, and 1% Pen/Strep. MDA‐MB‐231 cells were maintained in DMEM supplemented with 10% FBS and 1% Pen/Strep. FHC cells were maintained in DMEM/F12 supplemented with 10% FBS, 1% Pen/Strep, 20 ng/mL hEGF, 10 ng/mL cholera toxin, 5 μg/mL insulin, 5 μg/mL transferrin, 100 g/mL hydrocortisone, and 50 μM HEPES. All cells were cultured under a humidified atmosphere at 37°C and 5% CO_2_.

### 2.5. Cytotoxic Activity by MTT Assay

Cytotoxicity tests were performed in 96‐well plates. Cells were seeded at 8000 cells per well and allowed to attach overnight. STK extracts were first screened for cytotoxic activity at five concentrations, that is, 100, 50, 2, 12.5, and 6.25 μg/mL, against various cancer cell lines, including NCI‐H508, HCT15, HT29, HCT116, HepG2, HeLa, and MDA‐MB‐231. The noncancerous cell line FHC was used as a normal cell control. Doxorubicin was used as a positive control at a concentration of 2 μM. DMSO was used as a vehicle control and maintained at 0.5 %v/v final concentration in cell culture media for all test conditions. All experiments were done in triplicate. Cells were exposed to the test agents for 48 h. Then, to determine cell viability, the MTT assay was performed as previously published, and the methods description partly reproduces their wording [10]. Briefly, MTT solution was added into each well to a final concentration of 1 mg/mL and incubated for 4 h at 37°C. Afterward, the culture medium was aspirated, and DMSO was added at 100 μL per well. Absorbance at 550 nm was measured by the UV–Vis VICTOR Nivo Multimode Microplate Reader (PerkinElmer, USA).

### 2.6. Calculation of Selectivity Index

Selectivity index (SI) was calculated at 100 and 50 μg/mL based on the following equation:

(1) Selectivity index=100−Cell viabilitycancer cell100−Cell viabilityFHC.

### 2.7. Tumor spheroid Culture

Spheroid cultures were performed in 96‐well U‐bottom ultralow adherent plates as previously published, and the method description partly reproduces their wording [11]. Briefly, HCT116, HT29, and HepG2 cells were seeded at 200 cells/well and cultured for 2 d to allow spheroid formation. Spheroids were then exposed to 50 and 100 μg/mL of STK. DMSO was used as a vehicle control and maintained at 0.5% v/v final concentration for all test conditions. For each spheroid, pictures were taken every 2 d for 10 d (8 spheroids/treatment condition) with an inverted microscope (Eclipse TE 2000‐U, Nikon, Japan) under 40x magnification. To quantify spheroid size, the images were analyzed to measure their areas using the ImageJ software [12].

### 2.8. Flow Cytometry Analysis of Apoptotic Cell Death

Apoptosis was quantified via flow cytometry following a modified protocol based on previously published methods [11]. HT29, HCT116, and HepG2 cells were treated with test substances for 24 h. Cells were then harvested, and a suspension of 2 × 10^5^ cells per replicate was prepared in PBS. Cells were then stained with 5 μL of FITC‐conjugated annexin‐V in binding buffer (0.1 M HEPES, 1.5 M NaCl, 50 mM KCl, 50 mM MgCl_2_, 18 mM CaCl_2_, pH 7.4) for 15 min at room temperature in the dark. Propidium iodide (PI) was subsequently added to a final concentration of 5 μg/mL and incubated for another 15 min under the same conditions. Cells were analyzed with an Attune NxT flow cytometer (Thermo Fisher), and data were processed with Attune NxT Software (Thermo Fisher). All experiments were performed in triplicate.

### 2.9. Transwell Migration Assay

Transwell migration assay was performed in a 24‐well plate. HCT116 cells were seeded at 2 × 10^5^ cells in 300 μL serum‐free media into the upper compartment of the transwell insert. Five hundred microliters of media containing 20% FBS were transferred into each well, constituting the lower compartment. Cells were exposed to STK extract at 25 and 12.5 μg/mL. DMSO was used as a vehicle control and maintained at 0.5 %v/v for all treatment conditions. Cells were allowed to migrate for 40 h. Afterward, cells in the upper compartment were discarded with cotton swabs. Migrated cells were then fixed with 70% ethanol for 15 min. Membranes were then allowed to dry and then stained with 0.2% crystal violet. Afterward, membranes were washed to remove excess dye and allowed to dry. Pictures from 6 different microscopic fields were then taken with an inverted microscope (Eclipse TE 2000‐U, Nikon, Japan) under 40x magnification.

### 2.10. Statistical Analysis

Statistical significance was calculated using one‐way ANOVA with Tukey’s post hoc tests for Figures 1, 2, 3, S1, S3 and S5; while two‐way ANOVA with Tukey’s post hoc tests was used for Figures 4 and S4. GraphPad Prism software Version 7 was used for all statistical analyses. *p*‐value < 0.05 signified statistical significance.

Figure 1Cytotoxic effects of STK against CRC cell lines including (a) NCI‐H508, (b) HCT15, (c) HT29, and (d) HCT116. (e) FHC cells served as a control for noncancerous colonic epithelium. Doxorubicin 2 μM (dox) was used as the positive control. Each bar represents the mean ± SEM (*n* = 3). (^∗^ *p* < 0.05, ^∗∗^ *p* < 0.01 and ^∗∗∗^ *p* < 0.001 compared with the vehicle control [veh]).

**Figure:**
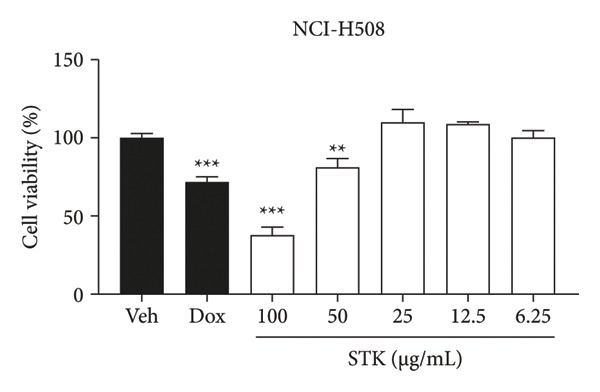
(a)

**Figure:**
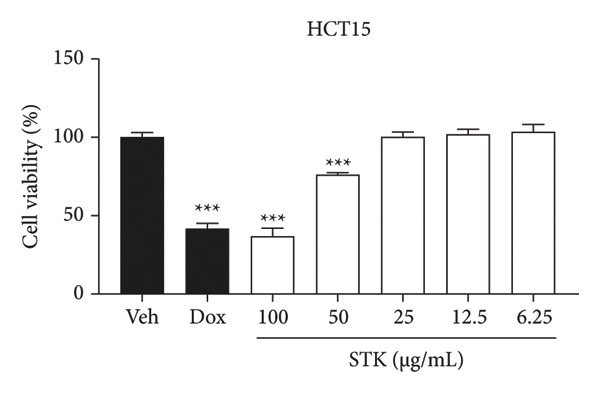
(b)

**Figure:**
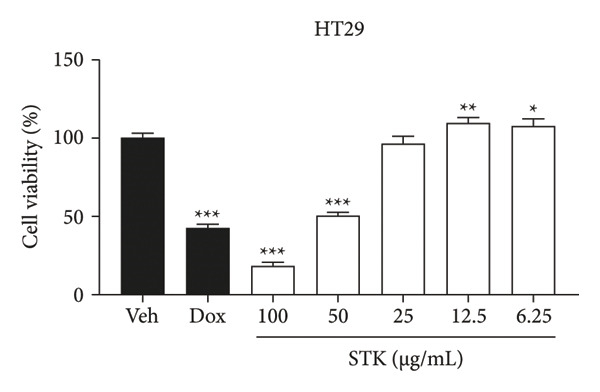
(c)

**Figure:**
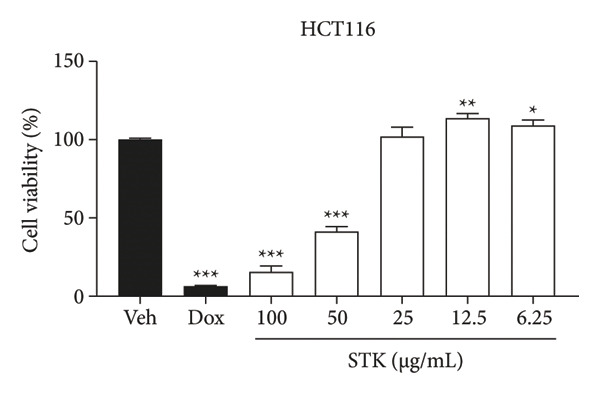
(d)

**Figure:**
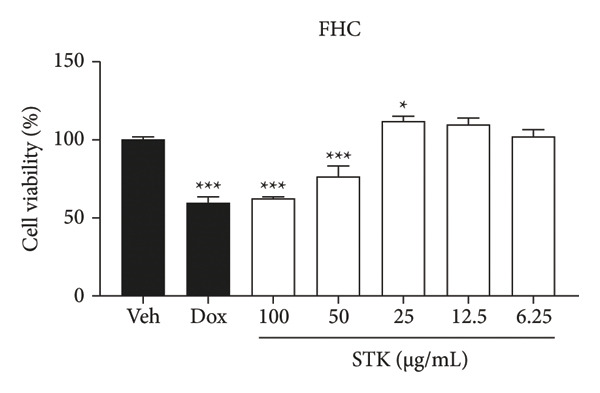
(e)

Figure 2Inhibitory effects of STK on colorectal tumor spheroid growth. Representative images of (a) HT29 and (d) HCT116 spheroids treated with vehicle (veh) or STK at concentrations of 50 μg/mL and 100 μg/mL over 10 days. Spheroid images were captured at Days 0, 2, 4, 6, 8, and 10. Quantitative analysis of (b) HT29 and (e) HCT116 spheroid size, normalized to the initial spheroid size at Day 0. Data are expressed as mean ± SEM (*n* = 8) with ● representing the vehicle control, ◼ representing STK at 50 μg/mL, and ◆ representing STK at 100 μg/mL. Area under the curve (AUC) analysis of spheroid size over the course of 10 days for (c) HT29 and (f) HCT116. (^∗∗∗^ *p* < 0.001 compared with the vehicle control (veh); ^###^ *p* < 0.001 compared with STK at 50 μg/mL).

**Figure:**
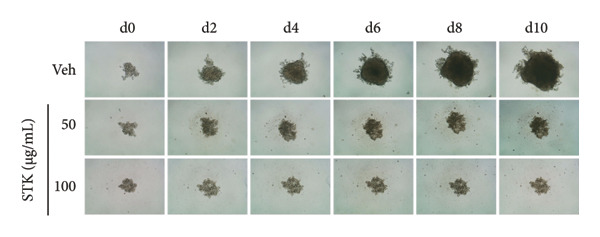
(a)

**Figure:**
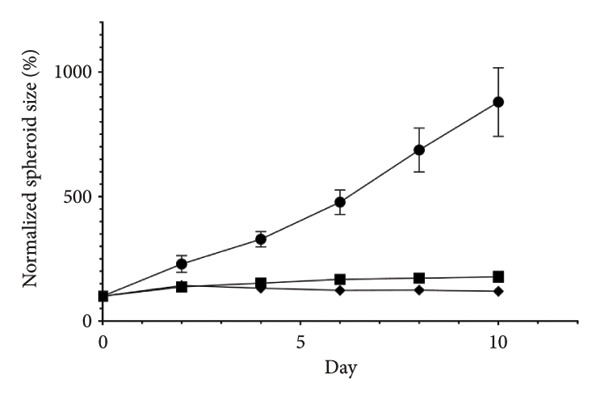
(b)

**Figure:**
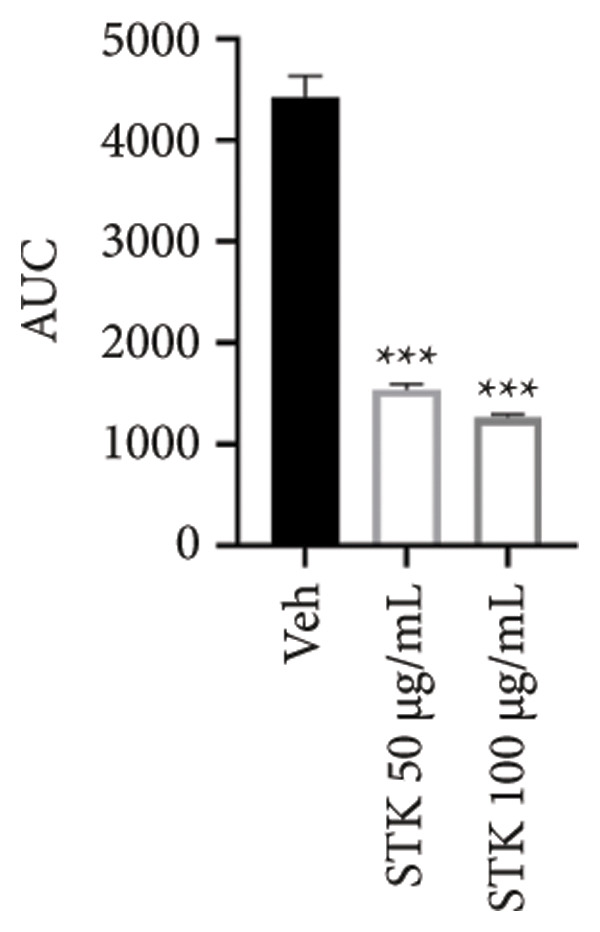
(c)

**Figure:**
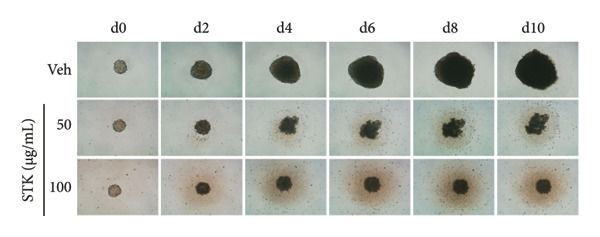
(d)

**Figure:**
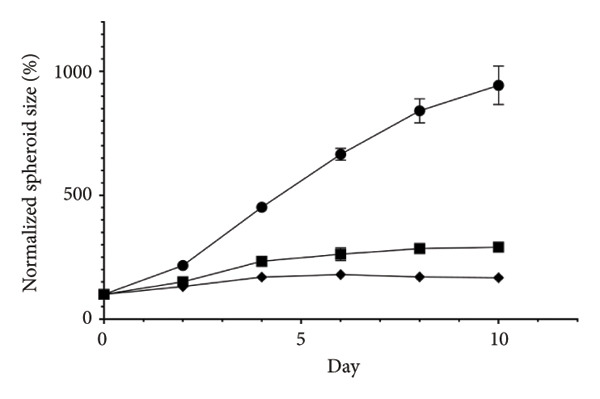
(e)

**Figure:**
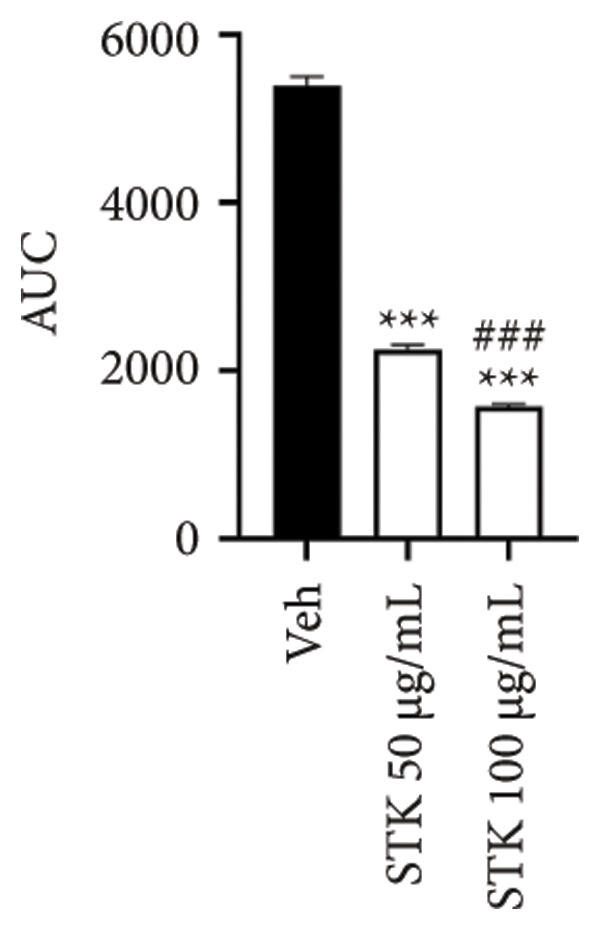
(f)

Figure 3Inhibitory effects of STK on transwell migration of HCT116 cells. (a) Representative images of migrated HCT116 cells stained with 0.2% crystal violet after treatment with the vehicle control (veh) or STK at concentrations of 12.5 μg/mL and 25 μg/mL. (b) Quantitative analysis of the number of migrated cells per treatment group. Data are expressed as mean ± SEM (*n* = 8). (^∗∗∗^ *p* < 0.001 compared with the vehicle control (veh); ^###^ *p* < 0.001 compared with STK at 12.5 μg/mL).

**Figure:**
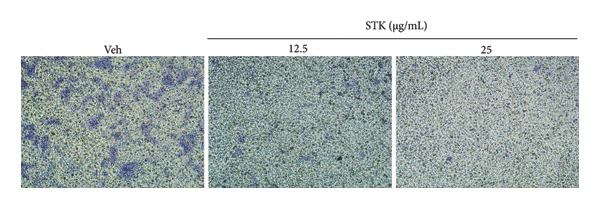
(a)

**Figure:**
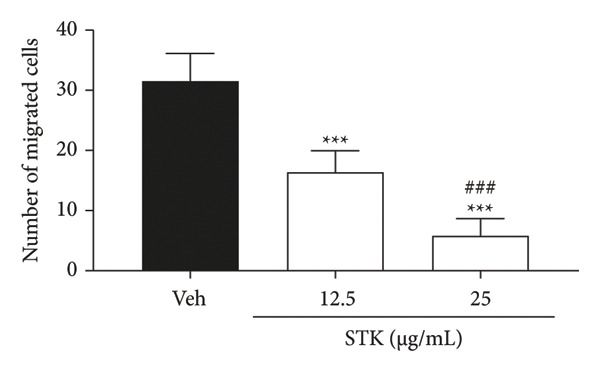
(b)

Figure 4STK treatment induced apoptotic cell death in CRC cell lines. Representative annexin‐V/PI flow cytometry plots of (a) HT29 and (c) HCT119 cells treated with STK for 24 h. Quantification of annexin V/PI staining for (b) HT29 and (d) HCT116. Data are expressed as mean ± SD (*n* = 3) ^∗∗^ *p* < 0.01 compared with the vehicle control (veh), ^∗∗∗^ *p* < 0.001 compared with veh, and ^###^ *p* < 0.001 compared with STK at 50 μg/mL.

**Figure:**
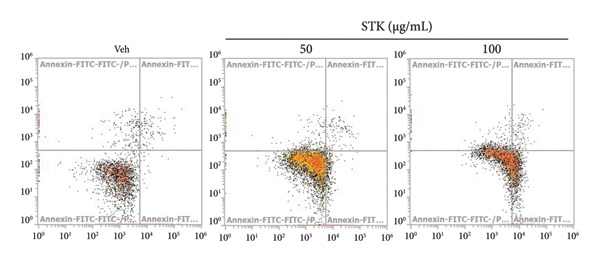
(a)

**Figure:**
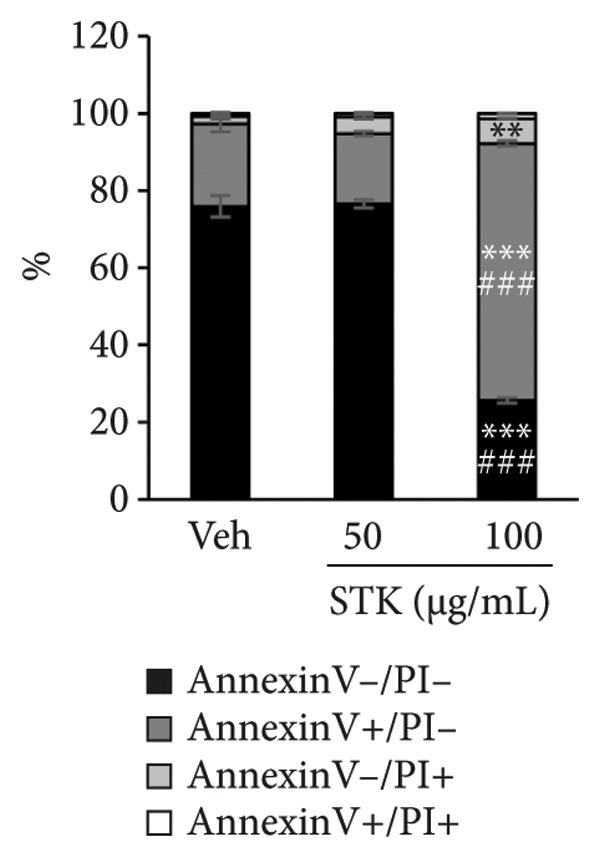
(b)

**Figure:**
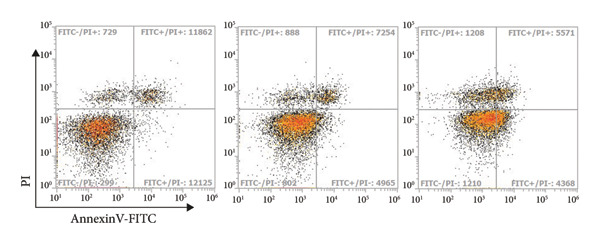
(c)

**Figure:**
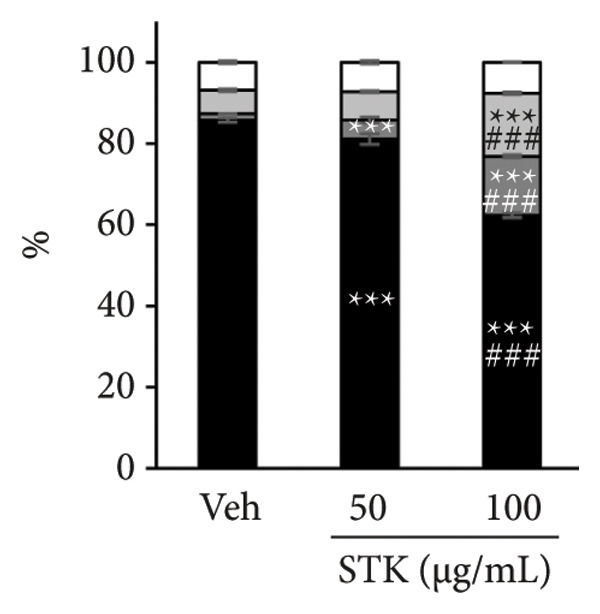
(d)

## 3. Results and Discussion

### 3.1. STK Suppressed the Viability of Cancer Cell Lines

In this study, four CRC cell lines, including NCI‐H508 (Figure 1(a)), HCT15 (Figure 1(b)), HT29 (Figure 1(c)) and HCT116 (Figure 1(d)) were utilized to investigate the cytotoxic effects of STK, while the FHC cell line (Figure 1(e)) served as a representative of normal, noncancerous cells. Doxorubicin, a widely used chemotherapeutic agent, was included as a positive control due to its well documented efficacy in targeting various cancer types. Indeed, STK significantly decreased the viability of cancer cells in a dose‐dependent manner (Figure 1). At high concentrations (50 and 100 μg/mL), STK markedly reduced cell viability across all CRC cell lines tested with statistical significance. A similar effect was observed in a hepatocellular carcinoma cell line, HepG2 (Figure S1A). Although some degree of cytotoxicity was detected against the noncancerous FHC cells, the inhibitory activity of STK at these concentrations was notably stronger (SI > 1) against gastrointestinal (GI) tract cancer cell lines when compared to FHC (Table 2), demonstrating an attractive in vitro efficacy and safety in line with its indication for GI tract malignancies. Additionally, STK also exhibited cytotoxic activity against non‐GI cancer, including HeLa (cervical, Figure S1B) and MDA‐MB‐231 (breast, Figure S1C), suggesting that it may hold potential against other cancer types, which should be further explored.

**Table 2:** Selectivity index of STK extracts against GI tract cancer cell lines.

Cell lines	Cell type	Cell viability		Cytotoxic activity (100‐viability)		SI	
		STK (μg/mL)		STK (μg/mL)		STK (μg/mL)	
		100	50	100	50	100	50
FHC	Colonic epithelium	62.67	76.78	37.33	23.22		
NCI‐H508	CRC	38.27	81.48	61.73	18.52	1.65	0.80
HCT15	CRC	37.44	76.61	62.56	23.39	1.68	1.01
HT29	CRC	18.83	51.33	81.17	48.67	2.17	2.10
HCT116	CRC	16.05	41.68	83.95	58.32	2.25	2.51
HepG2	Liver cancer	11.87	61.95	88.13	38.05	2.36	1.64

**Figure 5:**
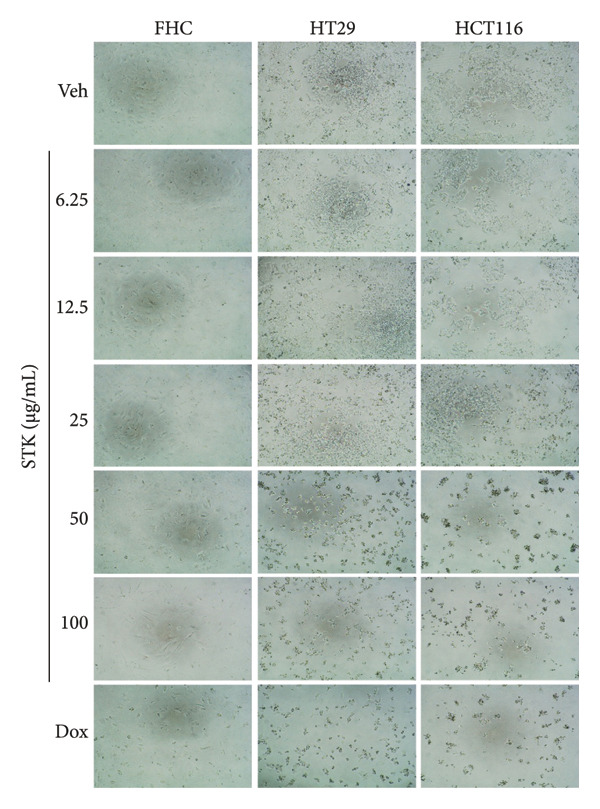
Representative images of morphological changes of HT29, HCT116, and FHC cells following STK exposure at the indicated concentrations for 48 h.

To further elucidate the impact of STK on CRC cell morphology, microscopic examination was performed following treatment with varying doses of STK or doxorubicin (Figure 5). The images revealed morphological alterations in cancer cell lines, characterized by reduced cell density and cell shrinkage, indicative of cytotoxic effects. A similar effect was observed in HepG2 cells (Figure S2).

### 3.2. STK Inhibited the Growth of Cancer Spheroids

To further validate the anticancer activity of STK and to better recapitulate the physiological condition, we sought to investigate the effects of STK on 3D tumor spheroid cultures, which are recognized as more physiologically relevant models than conventional monolayer cultures [13]. Microscopic images revealed that HT29 (Figures 2(a) and 2(b)) and HCT116 (Figures 2(d) and 2(e)) cancer cells readily form spheroids, which progressively increased in size over the course of 10 days. In line with our 2D data, STK treatment markedly suppressed the growth of HT29 (Figures 2(a) and 2(b)) and HCT116 (Figures 2(d) and 2(e)) tumor spheroids. Additionally, a similar effect was observed in HepG2 tumor spheroids (Figures S3A and S3B). Further analysis of the area under the curve (AUC) for spheroid size over time demonstrated that STK treatment significantly reduced AUC values compared to untreated controls (*p* < 0.001) (Figures 2(c), 2(f), S3C). Dose‐dependent suppression (*p* < 0.001) was observed in HCT116 (Figure 2(f)) and HepG2 (Figure S3C). However, in HT29 cells, no significant difference in spheroid suppression was observed between the two concentrations of STK (Figure 2(c)).

### 3.3. STK‐Induced Apoptotic Cell Death

To further characterize the observed cytotoxic activity, we performed flow cytometry to analyze apoptotic cell death in HT29 and HCT116 cells treated with STK. Indeed, a statistically significant increase in early apoptotic cells (Annexin V+, PI−) was readily detected at 100 μg/mL in both HT29 and HCT116 cells (Figure 4). A similar effect was observed in HepG2 (Figure S4). These data indicated that apoptosis was induced by STK treatment and support that the suppressed growth of tumor spheroids observed at the same concentrations (Figure 2) was likely mediated, at least in part, by increased apoptotic cell death.

### 3.4. STK Inhibited Migration of CRC Cell Line

Additionally, the effect of STK on cancer cell migration was evaluated using a transwell assay, with HCT116 cells chosen for their high metastatic potential. Microscopic images, visualized by 0.2% crystal violet staining, revealed the extent of cell migration (Figure 3(a)). The vehicle control group displayed a higher intensity of crystal violet staining, indicative of cell migration. STK‐treated groups showed noticeably reduced migration.

Quantitative analysis of migrated cells confirmed that treatment with low doses of STK (12.5 and 25 μg/mL) significantly inhibited migration compared to the vehicle control (*p* < 0.001) (Figure 3(b)). This inhibition was dose‐dependent, with the 25 μg/mL concentration exhibiting a significantly greater reduction in the number of migrated cells compared to the 12.5 μg/mL concentration (*p* < 0.001). These results suggest that STK effectively impairs cancer cell motility even at sub‐cytotoxic concentrations (Figures 1(d) and 1(e)), highlighting its potential as a therapeutic agent for limiting metastasis.

According to an interview with Panaran Phonphakdee, a traditional Thai medicine practitioner based in Nakhon Si Thammarat Province, he prescribed the Santhakhatpuakaln remedy primarily for GI malignancies, particularly liver and colon cancers. Remarkably, some early‐stage cancer patients using this remedy reportedly achieved a cure. For patients with advanced‐stage cancers, Santhakhatpuakaln has been credited with improving quality of life and, in some cases, extending survival. One notable case involved an 86‐year‐old patient with terminal colon cancer who was deemed ineligible for modern medical interventions, such as surgery or chemotherapy, due to advanced age and comorbid heart disease. After seeking treatment with STK, the patient experienced significant symptomatic relief, including reduced abdominal pain, normalized bowel movements, and an overall improved quality of life. Another compelling example is a Chinese patient diagnosed with terminal colon cancer and given a prognosis of only 5–6 months to live. After receiving traditional Thai medicine, primarily using STK, the patient lived for an additional 3 years before passing away. Beyond colon cancer, STK has also been used to treat other cancer types, such as breast cancer, further underscoring its potential as a complementary therapy in traditional Thai medicine. These anecdotal reports highlight the promise of STK, both its clinical efficacy and safety profile, warranting further scientific research to systematically evaluate its efficacy and mechanisms of action.

Our study provides compelling evidence supporting the anticancer properties of the traditional Thai remedy STK. Specifically, our findings demonstrated that STK extract exhibited significant cytotoxic effects against various cancer cell lines. Consistent with its traditional use, the most pronounced anticancer activity was observed against GI tract cancer cell lines, which displayed notably higher sensitivity to STK compared to the noncancerous FHC cell line. Moreover, the cytotoxic activity of STK was accompanied by the induction of cell apoptosis. Finally, the anticancer activity of STK was also replicated in our 3D tumor spheroid cultures, further validating its effectiveness in mimicking more physiologically relevant tumor models.

Another critical challenge in cancer treatment is metastasis, the expansion of cancer cells in organs located far from their original site of development, which has clinical implications [14, 15]. Interestingly, our study revealed that even at sub‐cytotoxic doses, STK significantly inhibited CRC cell migration in transwell assays, indicating its antimetastatic properties. This antimigratory effect could complement its cytotoxic activity, enhancing its overall therapeutic potential. Collectively, our data demonstrate that STK possesses robust anticancer properties, mediated through both cytotoxic and antimigratory mechanisms. Cumulatively, our findings fundamentally supported traditional medicinal use of STK and underscore the enduring value of Thai traditional medical heritage in modern cancer research.

Considering the ingredients of STK, as detailed in Table 1, several components have previously been reported to exhibit anticancer activities in studies conducted by various researchers. Notable plants with well‐characterized active compound(s) with extensive supporting evidence for anticancer activities include *A. dahurica* (Hoffm.) Benth. & Hook. f. ex Franch. & Sav. [16–18], *C. sativa* L. [19], *P. nigrum* L., and *Z. officinale* Roscoe [20].

The major constituents of the remedy are *A. marina* (Forssk.) Vierh. and *P. nigrum* L., each constituting ∼30% w/w in the recipe. Indeed, *P. nigrum* has been extensively studied for its anticancer properties across various experimental models, including breast cancer [21], CRC [22, 23], and cervical cancer [24]. The key bioactive compound responsible for these effects is piperine, which demonstrates anticancer activity through multiple mechanisms. These mechanisms include the induction of cell cycle arrest, promotion of apoptotic cell death, inhibition of cell proliferation, and suppression of cancer cell migration [25]. In contrast, the anticancer activity of *A*. *marina*, the other major component of the remedy, is not as well documented. Nevertheless, its ability to attenuate inflammatory responses in an animal model of ulcerative colitis has been reported [26]. This anti‐inflammatory effect could be particularly relevant to CRC treatment, as chronic inflammation is known to play a significant role in the pathogenesis and progression of this cancer type. By mitigating inflammation, *A*. *marina* may offer indirect therapeutic benefits in CRC.

## 4. Conclusion

Collectively, our findings underscore the potent anticancer properties of STK, as demonstrated by its ability to inhibit cell viability, apoptosis induction, suppress spheroid growth, and reduce cancer cell migration. Further research is essential to deepen our understanding of STK’s therapeutic potential. One promising approach is to identify the lead compound(s) within STK’s components through bioassay‐guided fractionation and small molecule purification. However, unlike modern medicine, which often relies on highly purified compounds for anticancer treatments, Thai traditional medicinal recipes commonly employ multiple ingredients synergistically to achieve a balanced effect. This holistic approach, rooted in folklore medicinal principles, aims to optimize therapeutic efficacy while minimizing side effects. Therefore, it is crucial to evaluate the anticancer properties of purified compound(s) in comparison to the whole or partially refined extract to determine whether the combinatorial effects of STK’s components are integral to its activity. Additionally, the mechanisms underlying STK’s anticancer effects and those of its active compounds represent a critical area for future investigation. Exploring STK’s potential in combination therapies could further enhance its therapeutic utility, particularly in contexts where it might complement existing chemotherapeutic agents. These avenues of research hold promise for translating STK’s traditional wisdom into scientifically validated strategies for cancer treatment.

## Conflicts of Interest

The authors declare no conflicts of interest.

## Funding

This work was financially supported by the Research and Creative Fund, Faculty of Pharmacy, Silpakorn University, awarded to Worrakanya Narakornwit. Purin Charoensuksai also acknowledges support for other projects from the Faculty of Pharmacy, Silpakorn University, and the Thailand Science Research and Innovation (TSRI) National Science Research and Innovation Fund (NSRF).

## Supporting Information

Supporting Table 1: Information on plant materials in the Santakatpuakaln remedy. Supporting Figure 1: Cytotoxic effects of STK against (A) HepG2, (B) HeLa, and (C) MDA‐MB‐231. Supporting Figure 2: Representative images of morphological changes of HepG2 cells following STK exposures at the indicated concentrations for 48 h. Supporting Figure 3. Inhibitory effects of STK on HepG2 tumor spheroid growth. (A) Representative images of HepG2 spheroids treated with vehicle (veh) or STK at concentrations of 50 μg/mL and 100 μg/mL over 10 days. Spheroid images were captured at Days 0, 2, 4, 6, 8, and 10. (B) Quantitative analysis of spheroid size over time, normalized to the initial spheroid size at Day 0. Data are expressed as mean ± SEM (*n* = 8) with ● representing the vehicle control, ◼ representing STK at 50 μg/mL, and ◆ representing STK at 100 μg/mL. (C) Area under the curve (AUC) analysis of spheroid size over the course of 10 days. (^∗∗∗^ *p* < 0.001 compared with the vehicle control (veh); ^###^ *p* < 0.001 compared with STK at 50 μg/mL). Supporting Figure 4: STK treatment induced apoptotic cell death in HepG2. (A) Representative Annexin‐V/PI flow cytometry plots of HepG2 treated with STK for 24 h. (B) Quantification of Annexin V/PI staining. Data are expressed as mean ± SD (*n* = 3) ^∗∗^ *p* < 0.01 compared with the vehicle control (veh), ^∗∗∗^ *p* < 0.001 compared with veh, and ^###^ *p* < 0.001 compared with STK at 50 μg/mL.

## Supporting information


**Supporting Information** Additional supporting information can be found online in the Supporting Information section.

## Data Availability

The data that support the findings of this study are available from the corresponding author upon reasonable request.
