# Supplementary material for: Potential Anticancer Activities of the Thai Traditional Medicinal Recipe Santakatpuakaln Against Colorectal Cancer Cell Lines
Source: Adv Pharmacol Pharm Sci. 2025 Oct 28;2025:6682780. doi: 10.1155/adpp/6682780 (PMC12562599; doi:10.1155/adpp/6682780)
Supplement: Supplementary file 1 — Supporting Information Additional supporting information can be found online in the Supporting Information section. [file ADPP-2025-6682780-s001.docx]

**Supplementary File**

**Potential anticancer activities of Thai traditional medicinal recipe Santakatpuakaln against colorectal cancer cell lines**

Worrakanya Narakornwit^1,2^, Roongtiwa Srisuphan^3^, Uthai Sotanaphun^2^, Pawaris Wongprayoon^1,4^, and Purin Charoensuksai^1,4^*

^1^Natural Products Research Center (NPRC), Faculty of Pharmacy, Silpakorn University, Nakhon Pathom, THAILAND

^2^Division of Industrial Pharmacy, Faculty of Pharmacy, Silpakorn University, Nakhon Pathom, THAILAND

^3^Faculty of Pharmacy, Silpakorn University, Nakhon Pathom, THAILAND

^4^Division of Biomedicine and Health Informatics, Faculty of Pharmacy, Silpakorn University, Nakhon Pathom, THAILAND

^*^Corresponding author Email: charoensuksai_p@su.ac.th, Tel: (+66)3 425 5800 ext. 208316, Fax: (+66)3 425 5800
